# Electrophoretic Mobility Shift Assay (EMSA) for Assessing RNA–Protein Binding and Complex Formation Using Recombinant RNA-Binding Proteins and In Vitro–Transcribed RNA

**DOI:** 10.21769/BioProtoc.5583

**Published:** 2026-06-20

**Authors:** David W. J. McQuarrie, Matthias Soller

**Affiliations:** 1 Department of Pharmacology, Weill Cornell Medicine, Cornell University, New York, NY, USA; 2 Division of Molecular and Cellular Function, School of Biological Sciences, University of Manchester, Oxford Road, Manchester, UK

**Keywords:** EMSA, Band-shift assay, RNA–protein complex, Multimerization, Kd determination, Protein complex, Recombinant protein expression, In vitro transcription

## Abstract

Evaluating RNA–protein interactions is key to understanding post-transcriptional gene regulation. Electrophoretic mobility shift assays (EMSAs) remain a widely used technique to study these interactions, revealing information about binding affinities and binding modalities, including cooperativity and complex formation. Here, we detail, in a step-by-step protocol, how to perform EMSAs. We describe how to generate, purify, and quantitate ^32^P-radiolabeled RNA by in vitro transcription, as well as the expression and purification of recombinant RNA-binding proteins in *E. coli* using ELAV as an example. We then describe how to set up binding reactions using serial dilutions in a microtiter plate format of recombinant ELAV and in vitro–transcribed RNA and how to perform EMSAs using native low-crosslinked acrylamide gels, with detailed graphically supported instructions and troubleshooting guides.

Key features

• Efficient production and purification of radiolabeled RNA probes via in vitro transcription and denaturing PAGE.

• Reproducible binding assays shown using recombinant ELAV protein as an example.

• Quantitative EMSA setup using serial dilutions in a microtiter plate for accurate binding curves.

• Native gel preparation and optimized running conditions for high-resolution separation of RNA–protein complexes.

## Graphical overview


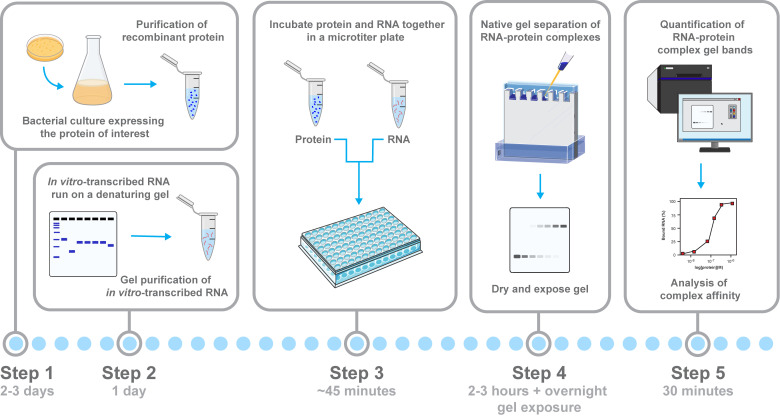


**Workflow for assessing RNA–protein binding and complex formation by electrophoretic mobility shift assays (EMSAs).** Schematic overview of the workflow for analyzing RNA–protein interactions using an EMSA, with relevant steps highlighted. The recombinant protein is expressed and purified from *E. coli*, and the ^32^P-radiolabeled RNA is generated by in vitro transcription and gel-purified. Then, protein serial dilutions are incubated with the RNA probe in a microtiter plate to allow for complex formation. Samples are separated on a native low-crosslinked acrylamide gel, where RNA–protein complexes show reduced mobility compared with free RNA. Key steps highlighted are protein purification, RNA preparation, binding setup, electrophoresis, and signal quantification.

## Background

RNA-binding proteins (RBPs) regulate multiple aspects of RNA metabolism, including splicing, polyadenylation, stability, localization, and translation [1–5]. Understanding how RBPs recognize specific RNA sequences or structures is key to uncovering post-transcriptional regulatory mechanisms. Electrophoretic mobility shift assays (EMSAs) remain a gold standard for analyzing direct RNA–protein interactions, as they allow the visualization of complex formation, binding modalities including cooperativity, and determination of binding affinities under near-physiological conditions. In addition, EMSAs are invaluable for mapping nucleic acid binding sites through mutagenesis or identifying key amino acid residues in RBPs that are essential for RNA binding.

The neuronal RBP ELAV (embryonic lethal/abnormal vision) from *Drosophila melanogaster*, homologous to human Hu family proteins, binds U-rich motifs and plays a critical role in many aspects of mRNA processing, including the inhibition of 3′ end processing leading to extension of 3′ UTRs, alternative splicing, and stabilization, localization, and translation [6–9]. Historically, ^32^P-radiolabeled RNA probes have been used in EMSAs due to their high sensitivity and easy quantification. Generally, RNA-ELAV/Hu or YTHDC RBP interactions in EMSAs have been highly reproducible [10–15], but the quality of both the RNA and protein components and consistent electrophoresis conditions are important.

This protocol provides instructions to generate and quantify high-purity ^32^P-radiolabeled RNA via in vitro transcription and gel purification, followed by native EMSAs using the recombinant ELAV protein. It includes detailed steps for gel preparation, binding reaction setup in a microtiter format, and critical handling of glass plates and buffers. The protocol is adaptable to other RBPs and can be used for other assays analyzing RNA–protein interactions, including footprinting assays, minimal binding site mapping, or analysis of large complexes [11,15–17]. We further provide a guide for troubleshooting and quantification of binding and complex assembly characteristics.

## Materials and reagents

**Biological materials**

1. *Escherichia coli* strain BL21(DE3) (e.g., NEB, C2527I) for the expression of GST- ELAV

**Reagents**

1. Tryptone (e.g., MilliporeSigma, catalog number: 16922)

2. Yeast extract (MilliporeSigma, catalog number: 70161)

3. Ampicillin (e.g., Sigma-Aldrich, catalog number: A9518)

4. Isopropyl β-D-1-thiogalactopyranoside (IPTG) (e.g., Sigma-Aldrich, catalog number: I6758)

5. Phosphate-buffered saline (PBS+), DEPC-treated and supplemented with 1 mM EDTA, 1 mM DTT, 5 μg/mL leupeptin prepared with DEPC-treated water, and 1 mM phenylmethylsulphonyl fluoride (PMSF) from 200 mM stock in isopropanol

6. Lysozyme (e.g., Sigma-Aldrich, catalog number: L7651)

7. Glutathione Sepharose 4B (e.g., Cytiva, catalog number: 17-0756-01)

8. NP-40/Igepal CA-630 (e.g., Sigma-Aldrich, catalog number: I3021)

9. PreScission protease (e.g., Cytiva/Amersham, catalog number: GE27-0843-01)

10. Protease inhibitor cocktail (Roche, Merck, catalog number: 4693116001)

11. High-quality glycerol (Invitrogen, catalog number: 1150874)

12. Diethyl pyrocarbonate (DEPC) to make RNase-free water (e.g., Thermo Fisher, catalog number: 10977015)

13. Liquid nitrogen

14. Phenol:chloroform:isoamyl alcohol (25:24:1) (e.g., Thermo Fisher, catalog number: 15593031)

15. Sodium acetate, pH 5.2 (e.g., Thermo Fisher, catalog number: AM9740)

16. Nucleotide mix (cold and hot NTPs, e.g., to label with α-^32^P ATP, ATP 0.2 mM, UTP 10 mM, GTP 10 mM, CTP 10 mM, final concentrations adjustable)

17. 10× transcription buffer (commercially supplied with T3/T7/SP6 polymerases)

18. Radiolabeled nucleotides (e.g., α-32P ATP or UTP, 800 Ci/mmol, 10 miCi/mL, 12.5 μM, PerkinElmer or Hartmann Analytics)

**CAUTION**: When handling radioactive nucleotides, always follow institutional radiation safety regulations.

19. RNasin Plus RNase inhibitor (e.g., Promega, catalog number: N2615)

20. RNA polymerase (T3, T7, or SP6) (Ambion)

21. DNase I (Ambion, catalog number: AM2222)

22. Dithiothreitol (DTT) (e.g., Thermo Fisher, catalog number: R0861)

23. Ethylenediaminetetraacetic acid (EDTA) (e.g., Thermo Fisher, catalog number: 15575020)

24. Ethanol (molecular biology grade) (e.g., Sigma-Aldrich, catalog number: E7023)

25. HEPES, pH 7.5 (e.g., Sigma-Aldrich, catalog number: H4034)

26. NaCl (e.g., Sigma-Aldrich, catalog number: S7653)

27. 2-Mercaptoethanol (optional, if used in sample buffers) (e.g., Sigma-Aldrich, catalog number: M6250)

28. Rain-X (Amazon) to silanize the inner plate for denaturing gels

29. Dishwashing liquid soap and 1 M KOH in methanol to wash EMSA plates

30. Tetramethylethylenediamine (TEMED) (e.g., Bio-Rad, catalog number: 1610801)

31. Ammonium persulfate (APS) (e.g., Sigma-Aldrich, catalog number: A3678)

32. Formamide (deionized) (e.g., Thermo Fisher, catalog number: AM9342)

33. Bromophenol blue (e.g., Sigma-Aldrich, catalog number: B0126)

34. Xylene cyanol FF (e.g., Thermo Fisher, catalog number: BP125)

35. Acetylated bovine serum albumin (BSA) (e.g., Thermo Fisher, catalog number: A2427)

36. Complete Mini EDTA-free protease inhibitor cocktail (e.g., Roche, catalog number: 11836170001)

37. pBluescript or pUC19 plasmid with RNA target sequence cloned downstream of T7/T3/SP6 promoter; pUC19 is used as a positive control in the NEB Gibson cloning kit (NEB, catalog number: E5520S)

38. Sodium dodecyl sulfate (SDS) (e.g., Thermo Fisher, catalog number: BP166)

39. TBE buffer, 10× or 5× (e.g., Thermo Fisher, catalog number: BP1333)

40. Tris-HCl, pH 7.5 (e.g., Sigma-Aldrich, catalog number: T5941)

41. tRNA (yeast, 10 mg/mL) (e.g., Sigma-Aldrich, catalog number: R5636)

42. 100 g acrylamide bottle (Bio-Rad, catalog number: 1610100)

43. Bis-acrylamide solution (Bio-Rad, catalog number: 1610142)

**Optional reagents**

1. Faustovirus capping enzyme (NEB, catalog number: M2081S)

2. Di-nucleotide cap (JENA Bioscience, catalog number: NU-854S)

**Solutions**

1. LB (Luria-Bertani) media (see Recipes)

2. LB agar (see Recipes)

3. CV buffer (cleavage buffer) (see Recipes)

4. Acrylamide/Bis-acrylamide solution (80:1) (see Recipes)

5. Formamide loading dye (blue juice) (see Recipes)

6. Cracking buffer (see Recipes)

7. Buffer A (see Recipes)

8. Buffer B (see Recipes)

**Recipes**

**1. LB media (per liter)**

10 g of tryptone

5 g of yeast extract

10 g of NaCl

**2. LB agar (per liter)**

LB media plus 15 g of agar

**3. CV buffer (cleavage buffer)**

50 mM Tris or HEPES pH 7.5 (at 25 °C)

150 mM NaCl

1 mM EDTA

1 mM DTT

**4. Acrylamide/Bis-acrylamide solution (80:1)**

In a 100 g acrylamide bottle, add 62.5 mL of 2% bis-acrylamide solution (or 1.25 g of bis-acrylamide powder if used) and fill up to 250 mL with DEPC water. Use a new 50 mL Falcon tube to measure the volume, using its grading line on top (the grading on the side is not correct). Avoid acrylamide powder in the air, as acrylamide is highly toxic.

**5. Formamide loading dye (blue juice)**

Add deionized formamide to 10 mg/mL bromophenol blue, 10 mg/mL xylene cyanol FF, and 5 mM EDTA.

**6. Cracking buffer**

Add 0.3 M sodium acetate (pH 5.2) in DEPC-treated water and add 0.2% SDS from the 20% stock.

**7. Buffer A**

Add 400 mM Tris or HEPES pH 7.5 (at 25 °C), 450 mM NaCl, 3 mM EDTA, 3 mM DTT, 0.25 mg/mL tRNA, and 0.5 mg/mL acetylated BSA.

**8. Buffer B**

Add 300 μL of CV buffer, 150 μL of 5× buffer A, and 300 μL of H_2_O (150 mM NaCl final).

## Equipment

1. Shaking incubator (e.g., Innova 44, New Brunswick)

2. Centrifuge (e.g., Sigma 3-30KS with swing-out rotor, 11134)

3. Microtip sonicator (XL, Heart Systems, model: XL2020)

4. Syringe and 0.45 μM filter (Millipore)

5. Perpex peristaltic pump, including tubing and connectors

6. Amersham Typhoon biomolecular imager (Cytiva or equivalent)

7. Scintillation counter (Beckmann or equivalent)

8. Gel tank (custom-made) and glass plates (20 cm × 20 cm inner plate, 20 cm × 22 cm outer plate) (e.g., Bio-Rad)

9. Spacers and combs (0.75 mm spacers and 20-well combs for EMSA, 1.5 mm spacers and 10-well combs for denaturing gel purification, 0.3 mm spacers and 20-well combs for analytical denaturing gel) (Bio-Rad)

10. Blu-tack (Amazon) to seal plates and tank

11. Powerpacks 200 V for EMSAs and 3,000 V for denaturing gels

12. EMSA gel loading tips (Alphalab, catalog number: LW1100R, 1–200 μL)

13. Denaturing gel loading tips (Corning, gel-loading pipette tips, catalog number: CLS4884, 1–200 μL, end of tip thickness 0.2 mm, tip flat)

14. Nanodrop or equivalent

15. 96-well clear V-bottom TC-treated microplate (Corning, catalog number: 3894)

## Software and datasets

1. Quantity One 1-D Analysis Software (Bio-Rad) or equivalent (e.g., ImageJ)

## Procedure

**A. Cloning of a tagged RNA-binding protein**

For ELAV, we used a modified pGEX vector (Addgene) to express a GST-ELAV fusion protein. We included a PreScission protease site to cleave GST from ELAV [TEV-protease (Promega) is an alternative]. If no antibody is available, it is advisable to include an epitope tag, e.g., FLAG (Rabbit F7425, Sigma) for ELAV at the N-terminus, as C-terminal fusions interfere with function [18]. Maltose-binding protein or His-tag fusions are alternatives. Also, dual tags can help with improving purity to avoid, e.g., ion exchange purification. If the protein is toxic to bacteria in pGEX because of leaky expression, T7-directed expression in an appropriate bacterial host is advisable [19]. Generally, we PCR-amplify protein coding regions from cDNA of tissue culture cells or *Drosophila* and clone them by Gibson assembly [20], but gBlocks (IDT) are an alternative. When doing mini-preps, we use the rest of the culture to IPTG-induce protein expression to check that positive clones also express protein on an SDS-protein gel stained with Coomassie.

**B. Culture and purification of recombinant protein from *E. coli***

1. Bacterial culture and induction of recombinant GST-HA-ELAV

a. Streak the GST-HA-ELAV construct onto LB-agar plates containing ampicillin.

b. Pick a single colony and inoculate a 5 mL starter culture in LB with ampicillin (1:1,000 dilution of 10% stock). Incubate overnight at 35.5 °C with shaking. Do not set the incubator at 37 °C, as the temperature will rise and induce a heat-shock response in bacteria that can be detrimental.

c. Pour the starter into 250 mL of LB with ampicillin (prewarmed to 35.5 °C) and grow to OD_600_ ~ 0.6 (approximately for 2–3 h) at 350 rpm for good aeration. Avoid overgrowing the culture.

d. Induce protein expression with IPTG to a final concentration of 0.2 mM (e.g., 50 μL from 1 M stock) and incubate for 4–6 h at room temperature with shaking.

2. Recombinant GST-HA-ELAV purification

a. Pellet cells by centrifuging at 800× *g* for 10 min. Remove the supernatant.

**Figure 1.**
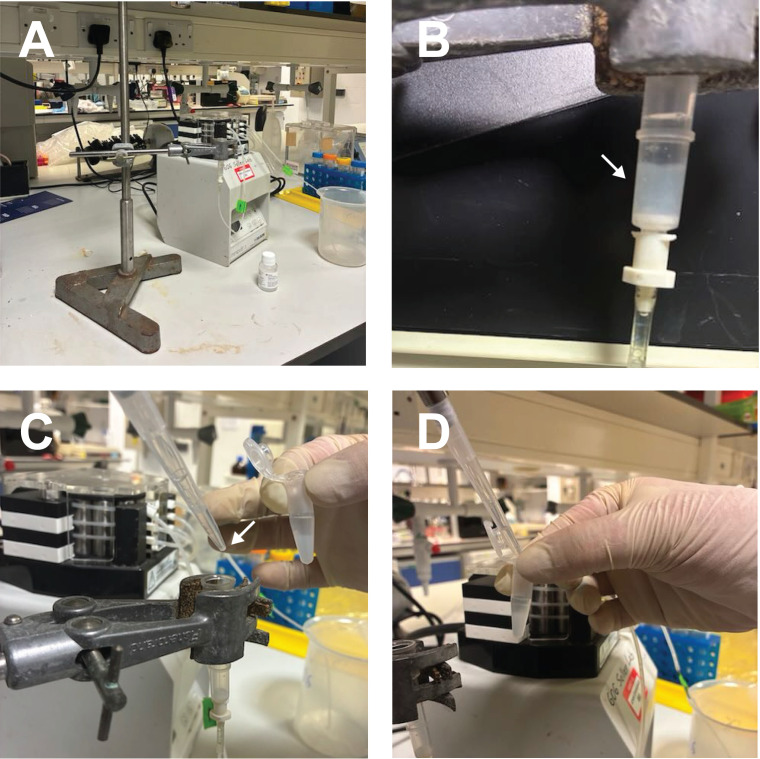
Example equipment and steps for purification of recombinant ELAV protein. (A) Peristaltic pump, polyprep column, and GST-tagged beads used for purification. The polyprep column has a mesh at the bottom to hold the GST beads. (B) Column after the addition of GST beads. The white arrow indicates the packed bead layer. (C) Removal of beads using a P1000 pipette tip with the end cut to facilitate extraction (white arrow). (D) Collected beads in an Eppendorf tube for subsequent washing or elution.

**Pause point**: Pellets can be stored at -80 °C until use.

b. Resuspend the bacterial pellet in 12.5 mL of chilled PBS+.

c. Add 250 μL of lysozyme (10 mg/mL) and incubate on ice for 15 min.

d. Sonicate on ice with a tip sonicator (e.g., 5–20 × 10 s bursts at medium power), without generating foam (foam is denatured protein; discard such samples).

e. Centrifuge at 15,000× *g* for 10 min at 4 °C in a swing-out rotor (e.g., Sigma 3–30KS).

f. Do not use a fixed-angle rotor, as residual RNases will co-purify.

g. Filter supernatant through a 0.45 μm membrane with a syringe. If filtering is difficult, centrifuge for longer.

h. Take samples from all steps to check protein expression and calculate amounts to exceed the capacity of the GST beads. PreScission protease is GST-tagged, and if bound to the beads, will not efficiently cleave ELAV.

i. Add 500 μL of glutathione Sepharose 4B slurry into a Bio-Rad polyprep column and equilibrate with 20 mL of PBS+ (Figure 1A, B). The GST bead amount can be adjusted to the amount of protein needed. Calculate the amount of protein present and ensure bead capacity is saturated.

j. Slowly load clarified lysate onto a Bio-Rad polyprep column. Wash with 3 × 10 mL of PBS+ or more if necessary. Connect to a Perpex peristaltic pump for continuous flow (Figure 1A).

k. Exchange buffer to cleavage buffer (see Recipes).

l. Transfer resin to an Eppendorf tube with cleavage buffer. Use a small volume to get the highest protein concentration. Most proteins will not precipitate at 10 mg/mL. Without NP-40, GST beads are very sticky. If NP-40 cannot be used, cut a 1 mL tip with an RNase-free scissor and pipette small amounts (Figure 1C, D).

m. Add 40 U PreScission Protease or less and incubate on a rotating wheel at 4 °C for 4 h. One unit is defined as the amount of enzyme needed to cleave 100 μg of fusion protein in 16 h to 90% completion at 5 °C in a buffer containing 50 mM Tris-HCl, pH 7.0, 150 mM NaCl, 1 mM EDTA, and 1 mM DTT.

n. Optionally, perform a second overnight cleavage to increase yield.

o. Remove supernatant, add 50 μL of washed beads to remove residual protease, and repeat until no beads remain.

o. Add 1× protease inhibitor (from 20× stock in DEPC water).

q. Measure protein concentration by absorbance at 280 nm using a spectrophotometer (1 cm pathlength, e.g., NanoDrop). For ELAV, 1 A_280_ = 2.47 mg/mL (ε = 20,650 M^-1^·cm^-1^; Mr = 51,019 Da), equivalent to 1 mg/mL = 0.4 A_280_. 1 μg = 19.6 pmol; 1 mg/mL = 19.6 μM; 1 μM = 51 μg/mL.

**EXAMPLE**:

r. If ELAV = 6.6 μg/μL, add 0.31 μL to 10 μL for a 4 μM solution.

s. If ELAV = 1 μg/μL, add 2.04 μL to 10 μL for a 4 μM solution. Typical yields: 1–3 μg/μL. Validate protein concentration and integrity on an SDS gel with different concentrations of a standard protein loaded (e.g., BSA, ovalbumin, or lysozyme) [21].

t. Store at 4 °C or flash-freeze aliquots at -80 °C. We do not add glycerol to the recombinant protein, as it might interfere with binding.

**C. Cloning of an RNA substrate**

Initially, we cloned the ELAV *ewg* binding site into pBluescript because it contains T7 and T3 polymerase promoters. However, an extra sequence will be present at the 5′ ends. More recently, we cloned the substrate RNA with a T7 promoter into pUC19. The only additional sequence then is the three Gs at the start required for efficient in vitro transcription. For short substrate RNAs, we have also used oligonucleotides for in vitro transcription [22]. In this case, only the T7 promoter needs to be double-stranded. However, the yield is generally lower than when cloned into a plasmid. For the selection of suitable RNA substrate sequences, we typically examine sequences from different species for evolutionary conservation [15,23].

**D. *In vitro* transcription, purification, and quantification of radiolabeled RNA**

1. Template plasmid preparation for in vitro transcription

a. Linearize 5–10 μg of plasmid using a restriction enzyme that does not generate a 3′ overhang (e.g., PstI) to avoid cryptic initiation.

b. Extract with phenol/CHCl_3_/isoamyl alcohol and precipitate in 0.3 M sodium acetate (pH 5.2, DEPC treated) and 1 μL glycogen (10 mg/mL) and three volumes of ethanol.

c. Dissolve the air-dried pellet in 10 μL of DEPC-treated water. To aid in dissolving the pellet, freeze once in liquid nitrogen and thaw.

2. In vitro transcription reaction setup

a. Prepare the nucleotide mix as described below. The hot (radiolabeled) to cold nucleotide ratio should be 5:20 μM in the final assembled transcription mix. Example for a 10× ATP mix (50 μL total):

CTP 10 mM, 5 μL (from 100 mM stock)

GTP 10 mM, 5 μL (from 100 mM stock)

UTP 10 mM, 5 μL (from 100 mM stock)

ATP 0.2 mM, 1 μL (from 10 mM stock)

H_2_O, 38 μL

**CRITICAL:** Adjust nucleotide concentrations depending on experimental requirements, e.g., for AU-rich sequences, reduce GTP and CTP to 2 mM. If capping is required, we recommend Faustovirus capping enzyme (NEB), but di-nucleotide caps can also be used (NU-854S, JENA Bioscience).

b. Assemble components in the following order to minimize precipitation of DNA and/or spermidine:

H_2_O, 2.5 μL

10× transcription buffer, 1 μL

10× NTP mix (ATP or UTP), 1 μL

α-^32^P-NTP (typical: 800 Ci/mmol, 10 mCi/mL, 12.5 μM), 3 μL

**CAUTION**: When working with radioactive nucleotides, always ensure that institutional safety rules are followed.

RNase inhibitor, 0.5 μL (20 U)

Linearized DNA template in DEPC-treated water (salt-free), 0.5–1 μg, 1 μL

RNA polymerase (T3, T7, or SP6), 20 U, 1 μL

c. Bring the total volume to 10 μL by adding the appropriate amount of DEPC-treated/RNase-free ddH_2_O.

d. Spin briefly and incubate at 37 °C for 60 min.

e. Add 1 μL of DNase I and incubate for 15 min. Then, add 0.5 μL of 0.5 M EDTA.

**Pause point:** Transcribed RNA can be stored at -80 °C for extended periods.

**Figure 2.**
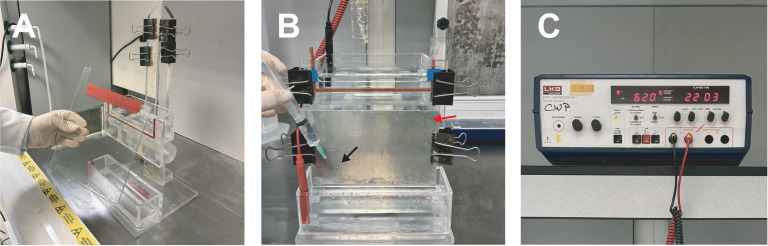
Urea gel preparation and running. Urea gel apparatus and directions, including the urea gel and gel tank with clips and spacers (A and B), and a high voltage power pack (C). Before loading, use a syringe with a bent hypodermic needle (B, black arrow) to remove the bubbles between the glass plates at the bottom. If large bubbles are present, the gel will run awkwardly or not at all, resulting in a short circuit/power failure or melting of the plastic tank. Denaturing gels are run at 50–60 °C to disrupt RNA secondary structure. The metal plate (B, red arrow) is essential to distribute the heat. Glass plates will crack in the absence of this aluminum metal plate. Analytical gels are thin using 0.3 mm spacers, here with a 32-sample comb to load a 1 μL sample.

3. Gel purification of in vitro–transcribed RNA

a. Pour a 4.5% denaturing urea polyacrylamide gel using silanized small plates for probes >100 nts using 1.5 mm spacers and a 10-well comb (Figure 2A). Adjust the percentage for smaller RNAs [24].

b. Dry the transcription reaction in a speed-vac, then add 20 μL of formamide loading dye (see Recipes).

c. Heat the mixture at 100 °C for 90 s and immediately place on ice.

d. Load samples onto a pre-run gel and run at 400–600 V at 50–60 °C until sufficient separation is achieved (Figure 2B, C).

e. Carefully dismantle the gel and remove the cover plate.

f. Wrap the gel in cling film (all plastic products are RNase-free from production unless touched by human skin, which is a strong source of RNases) and expose to autoradiography film for 20 s (adjust exposure as needed) in a dark room. Adjust one corner of the film to one corner of the glass plate to later localize the gel piece to cut out.

g. Mark the signal with a pen and put the developed film behind the glass plate to locate the RNA band to cut out. Cut the gel piece out as small as possible with a razor blade, and transfer to an Eppendorf tube (snap-cap; other tubes sometimes leak).

h. Re-expose the gel to confirm that the entire correct band has been excised.

i. Add 400 μL of cracking buffer (see Recipes) to the gel slice, repeatedly freeze in liquid nitrogen to improve RNA recovery, and incubate for at least 3 h or overnight on a rotating wheel.

j. Extract the supernatant with phenol/CHCl_3_/isoamyl alcohol and precipitate RNA with 1 μL glycogen (20 mg/mL) and 1 mL of ethanol. 

k. Dissolve the RNA pellet in 20 μL of 10 mM Tris, pH 7.5, containing 1 μL of RNase inhibitor. Freeze in liquid nitrogen to help dissolve.

**Pause point**: Store transcribed RNA at -80 °C for extended periods.

*Note: Radiolabeled RNA undergoes auto-radiolysis and loses signal, which should be considered when storing RNA.*

4. Quantification of in vitro–transcribed RNA

a. Dilute 0.5 μL of gel-purified RNA in 50 μL of RNase-free water.

b. Take 10 μL of this dilution and add to 1 mL scintillation fluid (corresponding to 0.1 μL of 20 μL in vitro transcript, see below for the activity).

c. Measure radioactivity in a scintillation counter according to the manufacturer’s instructions.

d. To calculate the incorporation rate, use the formula below. Typically, incorporation efficiency ranges from 5% to 40% (200 = dilution factor, 2 = conversion to dpm, 100 = percentage). The input is 3 μL of α-^32^P ATP (800 Ci/mmol, 10 miCi/mL, 12.5 μM), corresponding to 30 miCi (0.66 × 10^8^ dpm) on the reference date (adjust accordingly to Supplementary Table 1):

$$Incorporation\;\left(\%\right)=\;\frac{cpm\;after\;nucleotide\;removal\;\times \;200\;\times \;2\;\times \;100}{input\;dpm}$$

e. To calculate the amount of RNA synthesized, the cold input is 200 pmol (10 μL of 20 μM in the in vitro transcription reaction), and the radiolabeled input is 37.5 pmol (3 μL of 12.5 μM ATP in 10 μL reaction results in 3.75 μM); the total is 237.5 pmol for the labeled nucleotide. Now, adjust to the incorporation rate (Y pmol). Then, to calculate the amount of labeled RNA obtained, divide Y pmol by the number of ATPs in the RNA (if labeled with ATP), which yields the amount of RNA in pmol, typically 0.1–1.0 pmol.

$$Amount\;of\;RNA=\;\frac{Y\;pmol}{number\;of\;ATPs\;in\;the\;RNA}$$

**Example:**

- For a gel-purified in vitro transcript labeled with α-^32^P UTP from the *ewg* ELAV binding site construct BSAN, 60,117 cpm were measured as detailed above (0.1 μL of 20 μL, 4 days before reference date). Note that for labeling an in vitro transcript with UTP, the NTP concentrations above need to be changed to 10 mM ATP and 0.2 mM UTP.
- 60,117 × 200 × 2 × 100 divided by 660,00,000 × 1.214 (from Supplementary Table 1) = 30% incorporation.
- 30% of 237.5 pmol is 71.25 pmol (which is the gel-purified RNA).
- The *ewg* ELAV binding site construct contains 94 UTPs: 71.25 divided by 94 results in 758 fmol total amount, which, in 20 μL (see above), is 37.9 fmol/μL.

*Notes:*

*1. The RNA concentration used in EMSAs should be approximately 100-fold below the Kd. For ELAV, we use a concentration of 100 pM. Accordingly, a 10 μL reaction contains 1 fmol of RNA.*

*2. To set up a mix for 10 binding reactions, 0.26 μL of gel-purified BSAN RNA needs to be added to the 50 μL mix (see below).*

**E. EMSA with ELAV**

**Figure 3.**
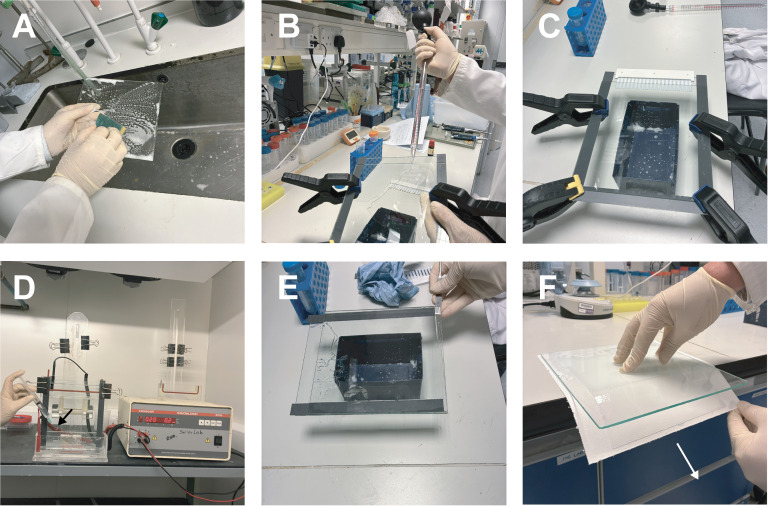
Electrophoretic mobility shift assay (EMSA) preparation and running. (A) Clean glass plates thoroughly with liquid soap before use and wash with ethanol. Then, wipe plates with KOH in methanol and wash thoroughly. This can help with opening the gel. (B) Dispense the EMSA polyacrylamide mix into the prepared gel case. (C) Insert the comb and leave the gel to polymerize horizontally. (D) Use a syringe fitted with a bent needle (black arrow) to carefully eliminate any air bubbles trapped at the base between the glass plates. Large bubbles can disrupt the electric field, causing uneven gel migration or, in severe cases, short circuits and melting of the electrophoresis tank. (E) Once the gel has run to the desired region, open the glass plates slowly. The gel should stick to one glass plate. (F) Place the gel on the filter paper and slide the plate and gel apart in the direction shown (white arrow).

1. Preparation of native gels for EMSAs

a. Clean glass plates thoroughly: rub with a sponge and liquid dish soap, rinse and dry with a paper towel, rub with 1 M KOH in methanol, rinse with water, and then rinse with ethanol (Figure 3A).

b. Do not silanize the plates (e.g., do not treat with Rain-X). Always use the same glass sides for EMSAs. The gels are very sticky. If the gel sticks to both sides, the experiment is generally ruined.

c. Prepare a 4% native polyacrylamide gel (3.5 mL of 40% 80:1, 3.5 mL of 5× TBE, 28 mL of H_2_O, 280 μL of 10% APS, and 14 μL of TEMED (Figure 3B, C).

**Optional**: A drop of glycerol in the gel mixture can help with reducing sticking to the glass plates when removing the gel.

d. Run gels in 0.5× TBE at 4 °C, 200 V. Pre-run for 60 min at 20 V and properly wash wells before loading. Set gel up before setting up the RNA-protein binding reactions (Figure 3D).

2. Preparation and loading of RNA–protein binding reactions

a. Use either freshly purified or flash-frozen recombinant ELAV without GST tag (-80 °C; stable for weeks at 4 °C with protease inhibitors; GST can dimerize, and results may become unpublishable).

b. Prepare a fresh 4 μM working dilution of ELAV in the original Eppendorf before use (e.g., 4 μL of H_2_O, 2 μL of 5× buffer A, plus ELAV in CV buffer to 4 μL). Always add ELAV last.

c. In a 96-well plate, prepare:

1 μM: 7.5 μL of buffer B + 2.5 μL of 4 μM ELAV – 2.5 μL to next well/discard 2.5 μL

0.25 μM: 7.5 μL of buffer B + 2.5 μL of 1 μM – 2.5 μL to next well/discard 2.5 μL

62.5 nM: 7.5 μL of buffer B + 2.5 μL of 0.25 μM – 2.5 μL to next well/discard 2.5 μL

15.6 nM: 7.5 μL of buffer B + 2.5 μL of 62.5 nM – 2.5 μL to next well/discard 2.5 μL

3.9 nM: 7.5 μL of buffer B + 2.5 μL of 15.6 nM – discard 5 μL

Control: 5 μL of buffer B only

d. Prepare RNA binding solution: x μL of RNA (as calculated above), 5 μL of tRNA (2 mg/mL), 1 μL of RNase inhibitor, and H_2_O to 50 μL. The RNA is in water to minimize the secondary structure [25,26].

e. Optionally, heat RNA to 60 °C for up to 5 min, then equilibrate at RT for 5 min.

f. Add 5 μL of RNA mix to each well containing protein dilutions and incubate for 15 min at RT.

g. Add 2 μL of 50% high-quality glycerol to an empty well (add dye-colored glycerol to input RNA).

h. Take 5 μL from each RNA–protein binding mix, add to the glycerol in the separate well by pressing down the pipette, and then take up the entire solution and load into the well.

3. Electrophoresis, drying, and exposure of EMSA gels

a. Run the gel in 0.5× TBE buffer at 4 °C at 200 V until separation is sufficient, e.g., the darker bromophenol dye is in the lower third.

b. Dismantle the gel such that the gel sticks to one plate (Figure 3E). Put a Whatman paper on top of the gel. The gel should now stick to the paper. Turn the gel face down and lift off the glass plate (Figure 3F). Put on the gel dryer and dry under vacuum at 80 °C for 1 h.

c. Expose to a phosphor screen overnight in a sealed cassette.

**Optional**: To minimize the risk of radiolabel contamination on the phosphor screen, a sheet of plastic film can be placed between the screen and the dried gel.

d. Image the phosphor screen using a Typhoon scanner.

4. Binding quantification and data analysis

a. Quantify bands with Quantity One 1-D software (Bio-Rad) or equivalent (e.g., ImageQuant or ImageJ).

b. Calculate the fraction of bound RNA as:

$$Fraction\;bound\;\left(\%\right)=\frac{Intensity\;of\;unbound\;RNA\;band}{Intensity\;of\;the\;0\%\;bound\;band\;from\;input\;RNA}\;\times 100$$

c. Plot the fraction bound against protein concentration and determine the apparent dissociation constant (Kd) by interpolating the protein concentration that results in 50% RNA binding (e.g., read from a generated graph) (Figure 4). Generally, we quantify the unbound RNA and calculate the bound fraction. Some proteins do not form a stable complex at certain RNA–protein ratios and fall apart while the gel is running. Accordingly, such a lane might look as if nothing is there because the label is distributed along the lane.

## Validation of protocol

This protocol has been recently used and validated in the following research article:

- McQuarrie, D. W. J., Soller, M. 2024. Phylogenomic instructed target analysis reveals ELAV complex binding to multiple optimally spaced U-rich motifs. *Nucleic Acids Research*.
  **Figure 4.**
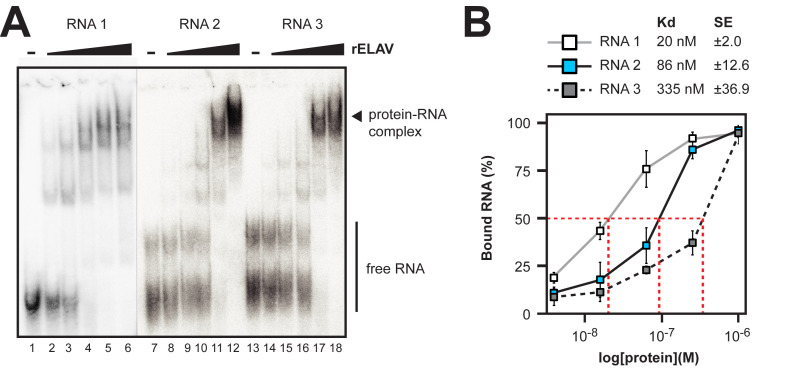
Example electrophoretic mobility shift assay (EMSA) gels and quantification of dissociation constants (Kds) for rELAV with synthetic RNAs. (A) Representative EMSA gel with RNA–protein complexes and free RNA labeled. Three RNAs were tested (RNA1, 2, and 3) for which sequence information is available in Supplementary Dataset 1. The free RNA and protein complex are labeled. A lack of visible protein complex formation on the gel would indicate no binding. (B) Plot of bound RNA at each protein concentration tested for RNA1 (white), RNA2 (blue), and RNA3 (grey). Calculated Kds and standard error (SE) are indicated above the plot. The red dashed lines indicate the 50% concentration point and the corresponding protein concentrations (Kd) for each RNA.

## General notes and troubleshooting

**General notes**

1. Always use RNase-free reagents, tips, and tubes for RNA work. Plastic is generally RNase-free from production. Do not autoclave tips; instead, use pre-racked tips.

2. Human skin is the major source of RNase. Always wear gloves that have not been in contact with human skin.

3. Keep recombinant proteins on ice and avoid repeated freeze-thaw cycles. Flash-freeze recombinant protein in liquid nitrogen.

4. Pre-calculate volumes and dilutions carefully to avoid errors in EMSA.

5. Use the same glass plates and glass slides consistently for EMSA to ensure reproducibility and separation of the gel from the glass plates.

6. When working with radioactive nucleotides, follow institutional safety rules. Generally, the label concentration is very low and should not require shielding, but use safety goggles for loading the gel, which is a critical step and should be done quickly.

7. EMSA gels are best when prepared in the evening before and stored at 4 °C for 2 h after polymerization. EMSA gels need to be run at 4 °C. Leave power supplies in the cold for at least one day before switching on to avoid disaster from condensed water in the power supply, which could generate a short circuit.

8. Our EMSA salt (NaCl) concentration is 75 mM, but this might need to be optimized for other proteins. Potassium or divalent cations such as Mg might be required.

9. As competitors for nonspecific binding, we use tRNA and acetylated BSA, but heparin is another option. Possibly, competitor concentrations need to be adjusted using a systematic approach.

10. Complexes should be loaded onto the EMSA gel upon reaching equilibrium, which might need systematic analysis of incubation times.

11. DEPC reacts with primary amines. Accordingly, Tris, HEPES, and EDTA cannot be treated with DEPC. Prepare with DEPC-treated water and adjust the pH by dipping on pH paper first, and then take aliquots to measure with the pH meter. The pH meter electrode cannot be RNase-free, so you cannot dip it in the solution.

12. As an alternative to band intensity–based calculations, Kd can also be estimated by fitting the binding data to a standard binding curve if users prefer.

**Troubleshooting**

1. If the transcription yield is low, check that the plasmid is linearized, that the T7 polymerase is active, and that nucleotide concentrations are optimal for your desired RNA sequence. Residual salt (e.g., NaAc from precipitation) inhibits in vitro transcription efficiently. Gel purification generally gets rid of abortive transcripts, but if necessary, adjusting nucleotide concentrations can help. Six or more Us in a row act as the termination signal for T7, T3, or SP6 polymerases [22].

2. Incomplete protease cleavage can depend on the incubation time or protease amount and likely needs adjustment according to the needs and costs of the protease.

3. Gel slots need to be extensively washed, as residual acrylamide in the slot disrupts RNA-ELAV complexes. Gels are generally pre-run for at least 1 h.

4. If encountering weak or smeared EMSA bands, optimize the gel percentage, pre-run time, buffer composition, and RNA:protein ratios. Additionally, validate recombinant protein stability via SDS gels and RNA integrity via denaturing urea gels.

5. Aggregation indicated by a high signal in the wells in EMSAs can be addressed by adjusting the concentration of tRNA and acetylated BSA as a competitor or including a small amount of non-ionic detergent such as NP-40. Adding 1 μL of heparin (mg/mL) before loading the gel can also reduce nonspecific binding but might also require systematic testing. To validate the specificity of interactions, cold competitor substrate RNA can be used. Antibodies can be used for supershift experiments to indicate specificity, but be aware that many antibodies, in particular polyclonal sera, can contain high levels of RNases. Placental RNase inhibitors (e.g., RNasin) inhibit RNase A-family enzymes, but not other RNases.

6. Binding and complex assembly times might need to be adjusted to reach equilibrium before loading the gel. A smear or absence of discrete bands indicates that the complex is unstable, but binding affinity can still be determined from the percentage of RNA present, where the free RNA runs on the gel.

7. We add 5 μL from the 10 μL binding mix into 2 μL of glycerol (high purity) in one go, press the handle of the pipette down, and take up all the solution without up- and down-pipetting to not disturb the RNA–ELAV protein complex. Avoid bubbles, as this results in disruption of the RNA–ELAV protein complex. We use recombinant ELAV protein without glycerol, which could interfere with binding. If glycerol interferes with binding, a 30%–60% sucrose solution might work.

8. We generally use 4% gels for EMSA, but the percentage can be adjusted for smaller or larger complexes. 4% EMSA gels are very sticky because of the low cross-linker concentration. Proper preparation of the glass plates is crucial to ensure the gel adheres to a single plate when separating them after electrophoresis. Experience in running gels helps. Wash plates immediately with soap and rinse with water. Then, washing with 1 M KOH/methanol, followed by extensive rinsing (check pH) generally helps. Silanizing glass plates is not necessary and, in fact, can be detrimental. 1 M KOH in methanol removes silane.

9. For separating very large complexes, a sand-blasted glass plate, together with a mixture of 3% acrylamide/0.66% agarose, can be used [27].

## Supplementary information

The following supporting information can be downloaded here:

1. Supplementary Table 1: ^32^P decay table
